# Chronic Oral Administration of Aluminum Hydroxide Stimulates Systemic Inflammation and Redox Imbalance in BALB/c Mice

**DOI:** 10.1155/2023/4499407

**Published:** 2023-10-10

**Authors:** Ana Beatriz Farias de Souza, Erika Tiemi Kozima, Thalles de Freitas Castro, Natália Alves de Matos, Michel Oliveira, Débora Maria Soares de Souza, André Talvani, Rodrigo Cunha Alvim de Menezes, Sílvia Dantas Cangussú, Frank Silva Bezerra

**Affiliations:** ^1^Laboratory of Experimental Pathophysiology, Department of Biological Sciences and Center of Research in Biological Sciences, Federal University of Ouro Preto (UFOP), Ouro Preto, MG 35402-136, Brazil; ^2^Laboratory of Immunobiology of Inflammation, Department of Biological Sciences, Institute of Exact and Biological Sciences, Federal University of Ouro Preto (UFOP), Ouro Preto, MG 35402-136, Brazil; ^3^Laboratory of Cardiovascular Physiology, Department of Biological Sciences and Center of Research in Biological Sciences, Federal University of Ouro Preto (UFOP), Ouro Preto, MG 35402-136, Brazil

## Abstract

The present study is aimed at investigating the long-term effects of the aluminum hydroxide administration in the small intestine, lung, liver, and kidney of male BALB/c mice. The mice received via orogastric gavage phosphate buffered or 10 mg/kg aluminum hydroxide 3 times a week for 6 months. Administration of aluminum hydroxide decreased hemoglobin, hematocrit, and erythrocyte. In the blood, kidney and liver function markers were evaluated, and long-term administration of aluminum hydroxide led to an increase in AST levels and a decrease in urea levels. The animals exposed to aluminum showed higher lipid and protein oxidation in all the organs analyzed. In relation to the enzymes involved in antioxidant defense, the lungs showed lower superoxide dismutase (SOD) and catalase activity and a lower reduced and oxidized glutathione (GSH/GSSG) ratio. In the liver, aluminum administration led to a decrease in catalase activity and the GSH/GSSG ratio. Lower catalase activity was observed in the small intestine, as well as in the lungs and liver. In addition to alterations in antioxidant defense, increased levels of the chemokine CCL-2 were observed in the lungs, lower levels of IL-10 in the liver and small intestine, and decreased levels of IL-6 in the intestine of the animals that received aluminum hydroxide for 6 months. Long-term exposure to aluminum promoted steatosis in the liver. In the kidneys, mice treated with aluminum presented a decreased glomerular density than in the naive control group. In the small intestine, exposure caused villi shortening. Our results indicate that long-term oral administration of aluminum hydroxide provokes systemic histological damage, inflammation, and redox imbalance.

## 1. Introduction

Aluminum (Al) occurs in different forms such as aluminosilicates, hydroxides, phosphates, sulfates, cryolite, and bauxite [1, 2]. Ertl and Goessler have reported that aluminum production has reached over 50 million tons in 2015 [3]. The physical and chemical characteristics of this metal allow for many industrial applications [4]. Besides its application in technology, the pharmaceutical industry has been using aluminum extensively as a component in cosmetics and in drugs such as antacids and aspirin [4].

Exposure to aluminum can happen through several routes, including the gastrointestinal and respiratory tracts. Human exposure to aluminum in 1950 was approximately 1 mg per day and will reach 100 mg per day by 2050 [4]. Dietary aluminum ingestion can vary from 1 to 20 mg per day [5]. However, the indiscriminate use of antacids could increase the aluminum intake to more than 500 mg per day [6]. The United Nations Food and Agriculture Organization (FAO)/World Health Organization (WHO) established the provisional tolerable weekly intake (PTWI) as 2 mg of aluminum per kilogram of weight [7]. Although studies have shown aluminum has low bioavailability, aluminum intake above the values recommended by FAO/WHO has toxic effects [4, 7].

The mechanisms involved in aluminum toxicity have not been described. However, previous evidence suggests that this metal has a pro-oxidant activity because of its capacity to form the aluminum superoxide radical AlO_2_^2+^ [4, 8]. Aluminum may also potentiate the inflammatory response, as it activates signaling pathways involved in the synthesis of inflammatory mediators [4]. Animals exposed for medium to long-term to aluminum-containing composites develop liver [9], kidney [10], intestine [11], and lung [12] injuries, besides being associated with microcytic anemia development [13].

Previous studies have evaluated the effects of aluminum chloride [9, 10, 12, 14] or aluminum oxide [15] intoxication. However, the effects of long-term use of aluminum hydroxide (Al(OH)_3_), a compound used to treat gastrointestinal diseases, have not been described. This medication does not require a physician's prescription, and indiscriminate use can have acute toxic effects [16, 17]. Thus, our hypothesis states that chronic ingestion of aluminum hydroxide would cause damage to several organs. Therefore, in the present study, we investigated the effects of long-term administration (6 months) of aluminum hydroxide in the small intestine, as well as the lung, liver, and kidney. We evaluated oxidative stress biomarkers, inflammatory mediators, and morphometric and histopathological parameters in these organs.

## 2. Materials and Methods

### 2.1. Animals

Eighteen (*n* = 18) male BALB/c mice, aged between 8 to 10 weeks and weighing between 28 to 30 grams, were obtained from the Federal University of Ouro Preto (UFOP) Animal Science Center. Animals received water and standard diet *ad libitum* and were conditioned under controlled temperature, light, and humidity (21 ± 2°C, 12 h light/dark, and 50 ± 10%, respectively). Experimental procedures followed the ethical principles established by the Brazilian Society of Science in Laboratory Animals (SBCAL) and were approved by the Ethics Committee on Animal Use (CEUA–UFOP) under protocol number 2017/05.

### 2.2. Exposure to Aluminum Hydroxide

The animals were randomly divided into three groups (*n* = 6): the control group (CG) that did not receive any type of intervention; the vehicle group (VG) that received via orogastric gavage, 200 *μ*L of phosphate-buffered saline (PBS) solution (pH 7.4); and the aluminum hydroxide (Al (OH)_3_) group (AHG) that received via orogastric gavage 10 mg/Kg of Al(OH)_3_ dissolved in 200 *μ*L of PBS [13]. The animals in the VG and AHG received orogastric gavage with the respective solutions three times a week for 6 months. Twenty-four hours after the last administration, the animals were anaesthetized with ketamine (130 mg/kg) and xylazine (0.3 mg/kg). The blood, bronchoalveolar lavage fluid (BALF), lungs, kidneys, liver, and small intestine were collected for analysis [18].

### 2.3. Blood Collection and Analysis

While the animals were under the effect of anesthetic, we collected their blood by cardiac puncture. We performed the euthanasia of the animals by exsanguination. Blood was used to evaluate the hematological and biochemical parameters and two aliquots were collected and placed in polypropylene tubes containing 15 *μ*L of anticoagulant (heparin, 5000 UI/mL). A 200 *μ*L aliquot was used to determine leukocytes, erythrocytes, hematocrit, hemoglobin, and platelets count, using an electronic counting device (Mindray Bio-Medical Electronics Co., Ltd., Shenzhen, China). From this aliquot, 2 *μ*L of blood was used to prepare blood smear slides that were stained with a rapid staining kit (catalog no. 620529, Laborclin, Pinhais, Paraná, Brazil). Subsequently, we performed a differential counting of leukocytes, differentiating the cells into monocytes, neutrophils, lymphocytes, and eosinophils [18, 19]. Two domain researchers counted unidentified slides at different times by double-blind counting.

The remaining collected blood was centrifuged at 10000 rpm for 15 minutes, and the plasma was collected and stored at -80°C. Subsequently, samples containing approximately 300 *μ*L of plasma were sent to the Pilot Laboratory of Clinical Analysis (LAPAC-UFOP) in order to determine the concentration of liver and kidney function markers, including aspartate aminotransferase (AST; K048-6), alanine aminotransferase (ALT; K049-6), alkaline phosphatase (ALP; K021-1), gamma-glutamyl transferase (GGT-1; K080-2), uric acid (K139-1), and urea (K056-1). The markers were determined by automatic spectrophotometry in the clinical analyzer Random Access Clinical Analyzer, Wiener Lab, model CM-200 (Wiener Lab, Rosario, Argentina) by the enzymatic-colorimetric method using specific kits (Bioclin®, Quibasa, MG, Brazil) [20].

### 2.4. Bronchoalveolar Lavage Fluid (BALF) Collection and Analysis

Immediately after euthanasia, the animals were tracheostomized with the aid of a catheter, and the right lung was perfused with 2 mL of saline solution (0.9% NaCl) for BALF collection. The samples were stored in polypropylene tubes and kept on ice (4°C) until the end of the experiment to prevent cell lysis. At the end of the experiment, the samples were centrifuged at 4°C, 3000 rpm for 10 min (MIKRO 200R; laboratory technology Hettich, Tuttlingen, Germany). The supernatant was stored, and the cells were resuspended in 0.1 mL of saline. Subsequently, 20 *μ*l of the resuspended solution was placed into a tube containing 180 *μ*l of Turk's solution, and the total leukocyte count was performed in a Neubauer chamber. For differential cell count of BALF, samples were centrifuged in a cyto-centrifuge (INBRAS health equipment, SP, BR). The slides were stained with a rapid staining kit (catalog no. 620529, Laborclin, Pinhais, Paraná, Brazil), and the leukocyte differential count was performed under an oil immersion optical microscope at 100x magnification [21]. Cell counting was performed by 2 researchers at different times by double-blind counting.

### 2.5. Organ Collection

After the BALF collection, the thorax of the animals was opened, and the left ventricle was perfused with saline solution (0.9%) to remove excess blood from the organs. Subsequently, the lung, liver, kidneys, and small intestine were collected. Approximately, 100 mg of each tissue was placed in polypropylene tubes and homogenized with buffer solution (pH 7.8). Then, the samples were centrifuged at 13000 rpm for 10 minutes at 4°C (MIKRO 200R; laboratory technology Hettich, Tuttlingen, Germany). The supernatant was collected and stored at -80°C for biochemical analyses [21].

The left lung, left kidney, and left lobe of the liver were immersed in 4% buffered formalin solution for 48 hours. Tissues were dehydrated, cleared, embedded in paraffin, cut into 4-5 *μ*m sections, and stained with hematoxylin and eosin (H&E). The entire small intestine was separated from the mesentery, washed in PBS at 0.01 M, pH 7.3, and extended to serosa in contact with the filter paper [22]. The antimesenteric border was opened, and all its contents were removed without damaging the mucosa. These fragments were immersed in the Bouin solution with 2% glacial acetic acid during 10 minutes. The prefixed intestine was rolled into a spiral with the mucosa facing inward to form rollers from the distal portion (posterior intestine) to the proximal portion (median intestine), which is an adaptation of the technique described by Calvert et al. [23]. The intestine rolls were fixed in 10% buffered formalin solution, processed, and stained with H&E.

### 2.6. Analysis of Oxidative Stress Biomarkers

In order to evaluate the oxidative effects from the aluminum hydroxide administration, the tissues of the lung, liver, kidney, and small intestine were analyzed. The superoxide dismutase (SOD) activity was determined as described by Marklund S. and Marklund G., which is based on the ability of SOD to inhibit pyrogallol autooxidation [24]. Catalase (CAT) activity was determined according to the method described by Aebi, which is based on the enzymatic breakdown of hydrogen peroxide over a 60-second interval by spectrophotometry [25]. The glutathione analysis was adapted from Sigma's commercial kit # CS0260, which uses a kinetic method to measure total glutathione (GSH+GSSG) levels in biological samples by reducing 5,5′-dithio-bis-(2-nitrobenzoic acid) to 5-thio-2-nitrobenzoic acid. To measure the levels of oxidized glutathione (GSSG), the biological samples went through a derivatization process using vinylpyridine and triethanolamine. Subsequently, the concentration of oxidized glutathione in the samples was determined based on a standard curve. The concentration of reduced glutathione (GSH) in the samples was obtained by subtracting the oxidized glutathione value from the total glutathione value. The GSH/GSSG ratio was obtained by dividing the GSH concentration results by the GSSG [26].

In order to measure lipid peroxidation levels, the method described by Buege and Aust was used, which is based on the ability of thiobarbituric acid to bind to oxidized lipids [27]. The determination of protein carbonylation levels was performed based on a protocol adapted from the methodology described by Reznick and Packer [28]. In addition, for the lung samples, the myeloperoxidase enzyme activity was determined by the spectrophotometric method at 630 nm [29]. In all the samples, the determination of total protein levels was measured according to the Lowry method [30].

### 2.7. Immunoassays for Inflammatory Markers

The tissue homogenate was used for the analysis of monocyte chemotactic protein-1 (MCP-1 or CCL2), interleukin 6 (IL-6), and interleukin 10 (IL-10). Assays were performed in 96-well plates, and 100 *μ*L of monoclonal antibody against the peptide (or proteins) reconstituted in PBS was added. After 12 hours of incubation at room temperature, blocking was performed with a solution containing PBS and 1% fetal bovine serum for two hours. Samples were added in a volume of 25 *μ*L per well. Subsequently, secondary antibodies diluted in PBS and 1% fetal bovine serum were added. The intensity of staining was read in an ELISA reader at 450 nm. Quantification of the inflammatory markers present in the samples was determined based on the optical density obtained with the standard curve of known protein concentrations [18, 31].

### 2.8. Morphometric and Histopathological Analysis

For the morphometric and histopathological analyses, the slides, stained with H&E, were photographed using the Primo star light microscope equipped with the Axiocam 105 digital camera (Carl Zeiss AG, Oberkochen, Germany) coupled with the ZEN lite image capture software.

In order to evaluate the structure of the lung parenchyma, twenty random fields were photographed using 40x magnification to obtain uniform and proportional lung samples. The images obtained were analyzed for volume density of alveolar septa (Vv [sa]) and alveolar spaces (Vv [a]). The total area of 1.94 mm^2^ was analyzed to determine the volume density of the alveolar septa (Vv [sa]) and the alveolar spaces (Vv [a]) as described by Mandarim de-Lacerda and Kozima et al. [18, 32].

Liver slides were photographed using 40x magnification to analyze liver steatosis and inflammatory reaction. Fat degeneration and inflammation were graded according to the percentage of fat-containing hepatocytes and the presence of focal inflammatory reaction, respectively. For this purpose, a grid composed of 100 squares (representing 100%) was overlayed on the images, and a percentage was assigned according to the number of squares in which steatosis and inflammatory reactions were observed. Subsequently, a score ranging from 0 to 4: (0) none, (1) 1–9%, (2) 10–33%, (3) 34–66%, and (4) more than 67%, was assigned [33]. In order to assess the total number of inflammatory cells in the lobules, including resident Kupffer cells, ImageJ/Fiji 1.46r software (Wayne Rasband, National Institute of Mental Health, Maryland) was used on a total area of 692300 *μ*m^2^. There were twenty-five random photomicrographs taken using 100x magnification with immersion oil for differential counting of mononuclear and polymorphonuclear cells [29].

The H&E-stained kidney slides were photographed using 4x magnification. The images at 4x magnification were used for the analysis of renal glomerulus, for which ImageJ/Fiji 1.46r software was used. The renal glomerulus area was considered the area bounded by the capillary tuft, and the capsular cavity area was determined by the renal corpuscle area subtracted from the renal glomerulus area [34]. Glomerular density was determined by calculating the total number of glomeruli considered histologically normal divided by the total area of the renal cortex [34, 35].

Histological sections of the jejunum were analyzed using the 10x objective [36, 37]. The obtained photomicrographs were quantitatively analyzed using ImageJ/Fiji 1.46r software. The height of 15 villi (Vh), the depth of 15 adjacent crypts (Cd), and the area of 15 villi were obtained. In order to assess the degree of intestinal injury, the villus height/crypt depth ratio (Vh/Cd) was calculated; for this, the villus height was divided by the depth of the adjacent crypt.

### 2.9. Statistical Analyses

Data were expressed as mean ± standard deviation. The evaluation of data normality was performed using the Kolmogorov–Smirnov test. One-way ANOVA followed by Tukey's post hoc test was used for parametric data. The Kruskal-Wallis test followed by Dunn's post hoc test was used for nonparametric data. The difference was considered significant when *p* < 0.05. All analyses were performed using GraphPad Prism software version 5.00 for Windows 7 (GraphPad Software, San Diego, CA).

## 3. Results

### 3.1. Evaluation of Aluminum Hydroxide Administration on Hematological Parameters

The analysis of the hematological parameters is presented in Table 1. Animals that received aluminum hydroxide had lower erythrocyte count (ANOVA, *F* = 19.45, *p* = 0.0002) and hemoglobin concentration (ANOVA, *F* = 7.51, *p* = 0.0055) compared to the naive control group (CG; *p* = 0.01). In the hematocrit (ANOVA, *F* = 11.48, *p* = 0.0009) and platelet count parameters (ANOVA, *F* = 12.98, *p* = 0.3656), the AHG showed lower levels compared to CG and VG (*p* < 0.05). We did not observe any statistical difference in total and differential leukocyte counts in the blood (*p* > 0.05) (Table 1).

We have also evaluated markers for liver and kidney function in the plasma. AST (ANOVA, *F* = 2.70, *p* = 0.0009) levels were higher in the animals that received aluminum hydroxide compared to the CG and VG (*p* = 0.01). In addition, the animals in the AHG had lower levels of urea (ANOVA, *F* = 7.81*p* = 0.0067) compared to the naive control group (CG; *p* = 0.01) (Table 1).

### 3.2. Cell Recruitment to Bronchoalveolar Lavage Fluid

**Figure 1:**
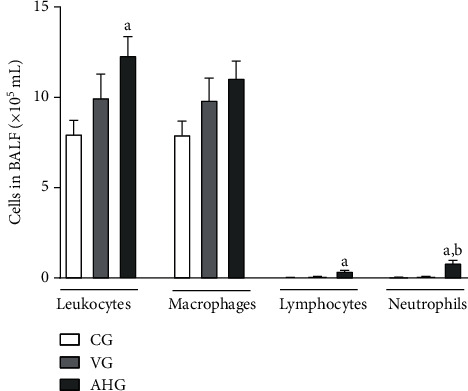
Total and differential bronchoalveolar lavage fluid cell count. CG: control group; VG: vehicle group; AHG: aluminum hydroxide group. The letter (a) represents a significant difference between groups when compared to CG. Data were expressed as mean ± standard deviation or median and interval between quartiles and were analyzed by one-way ANOVA or Kruskal-Wallis followed by Tukey or Dunn's posttest, *n* = 6 animals per group.

As shown in Figure 1, the animals in the AHG (12.33 ± 2.50) showed higher leukocyte count (ANOVA, *F* = 4.34, *p* = 0.0324) compared to the naive control group (CG; 8.0 ± 1.78) (*p* < 0.05). In addition, the lymphocyte (Kruskal-Wallis, *p* = 0.0068) count in the group exposed to aluminum hydroxide (0.4 ± 0.25) compared to CG (0.01 ± 0.02) (*p* < 0.05). Neutrophil recruitment (ANOVA, *F* = 16.37, *p* < 0.0001) in AHG (0.85 ± 0.47) was higher compared to CG (0.04 ± 0.04) and VG (0.07 ± 0.01) (*p* = 0.001) (Figure 1).

### 3.3. Oxidative Stress Biomarkers in the Lung, Liver, Kidney, and Small Intestine

As shown in Table 2, in the lung, long-term administration of aluminum hydroxide promoted a lower activity of the SOD (ANOVA, *F* = 5.09, *p* = 0.0205) and catalase (ANOVA, *F* = 3.66, *p* = 0.0504), as well as a lower ratio of GSH/GSSG ratio (Kruskal-Wallis, *p* = 0.0063) in the lung compared to the naive control group (CG; *p* < 0.05). Myeloperoxidase oxidant enzyme activity (ANOVA, *F* = 3.70, *p* = 0.0491) in the lung was higher in the AHG compared to the naive control group (CG; *p* < 0.05). The AHG presented higher concentration markers of lipid peroxidation (ANOVA, *F* = 8.29; *p* = 0.0037) and protein oxidation (ANOVA, *F* = 14.19, *p* = 0.0003) compared to CG and VG (*p* = 0.01) (Table 2).

In the liver, catalase activity (ANOVA, *F* = 9.48, *p* = 0.0022) and the GSH/GSSG ratio (Kruskal-Wallis, *p* = 0.0388) were lower in the group exposed to aluminum hydroxide compared to the naive control group (CG; *p* < 0.05). The levels of lipid peroxidation (ANOVA, *F* = 9.55, *p* = 0.0021) and carbonylated protein (ANOVA, *F* = 24.03, *p* < 0.0001) were higher in AHG compared to CG and VG (*p* = 0.01). In the kidney, we did not observe any difference between the experimental groups for antioxidant enzyme activity. However, the group exposed to aluminum hydroxide showed higher levels of lipid peroxidation (ANOVA, *F* = 5.02, *p* = 0.0213) and protein carbonylation (ANOVA, *F* = 12.51, *p* < 0.0001) compared to CG and VG (*p* < 0.05). In the small intestine, similar to the observations in the lung and liver, the catalase activity (ANOVA, *F* = 6.51, *p* = 0.0092) was lower in AHG compared to CG (*p* < 0.05). Furthermore, as observed in the other organs, the animals exposed to aluminum hydroxide showed elevated levels of protein (ANOVA, *F* = 41.71, *p* < 0.0001) and lipid oxidation (ANOVA, *F* = 8.99, *p* = 0.0027) when compared to CG and VG (*p* = 0.01).

### 3.4. Inflammatory Markers in the Lung, Liver, Kidney, and Small Intestine

As shown in Table 3, in the lung homogenate, CCL-2 level (ANOVA, *F* = 8.57, *p* = 0.0049) was increased in the aluminum hydroxide group compared to naive control group (CG; *p* = 0.01). In the liver, IL-10 levels (ANOVA, *F* = 5.01, *p* = 0.0263) were lower in AHG compared to the naive control group (CG; *p* < 0.05). In the small intestine, long-term administration of aluminum hydroxide resulted in lower levels of IL-10 (ANOVA, *F* = 4.44, *p* = 0.0359) and IL-6 (ANOVA, *F* = 4.44, *p* = 0.0359) compared to the CG (*p* < 0.05). No differences were observed between the experimental groups for the cytokines evaluated in the kidney (Table 3).

### 3.5. Histopathological and Morphometric Analyses of the Lung, Liver, Kidney, and Small Intestine

**Figure 2:**
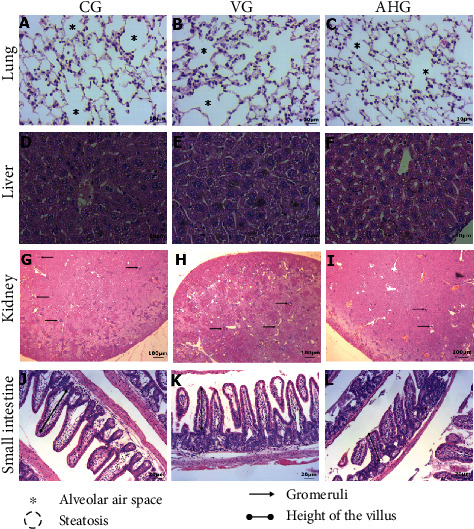
Photomicrographs of lung, liver, kidney, and small intestine sections stained with hematoxylin and eosin. CG: control group (A, D, G, J); VG: vehicle group (B, E, H, K); AHG: aluminum hydroxide group (C, F, I, L). Photomicrographs of lung sections (A–C), bar = 50 *μ*m; asterisks (^∗^) indicate alveolar airspace area. Photomicrographs of liver sections (D–F), bar = 50 *μ*m; in (F), the circle marks regions of steatosis. Photomicrographs of kidney sections (G–I), bar = 100 *μ*m; the thin arrows indicate glomeruli. Photomicrographs of small intestine sections (J–L), bar = 20 *μ*m.

The histopathological and morphometric analyses are presented in Figure 2 and Table 4. Regarding the stereological analyses of the volume density of alveolar airspace (Vv [a]) and the volume density of alveolar septal (Vv [sa]), there was no difference between the experimental groups (Table 4, Figures 2(A)–2(C)). In the liver, histopathological evaluation demonstrated that aluminum hydroxide administration resulted in vacuolization of hepatocytes. Quantitative analysis showed a higher score for steatosis (Kruslkal Wallis, *p* < 0.0001) in AHG livers compared to CG and VG (*p* < 0.0001) (Table 4, Figures 2(D)–2(F)). Furthermore, there was no increase in inflammatory foci in the liver parenchyma of AHG (*p* > 0.05).

Glomerular density analysis (ANOVA, *F* = 6.027, *p* = 0.015) demonstrated that long-term aluminum hydroxide administration promoted a decrease in the number of glomeruli in the renal parenchyma with the naive control group (CG; *p* < 0.05) (Table 4, Figures 2(G)–2(I)). The aluminum hydroxide group showed a shortening of the villi in the intestine compared to the other groups. The quantitative analyses showed a decrease in villus height (ANOVA, *F* = 7.90, *p* = 0.0034) and a reduction in the ratio between villus height and crypt depths (Vh/Cd) (Kruslkal-Wallis, *p* = 0.0009) compared to CG and VG (*p* < 0.05). In addition, AHG showed smaller villus areas (Kruslkal-Wallis, *p* < 0.0061) compared to the naive control group (CG; *p* = 0.01) (Table 4, Figures 2(J)–2(L)).

## 4. Discussion

In the present study, we showed that oral administration of aluminum hydroxide for 6 months causes inflammation and oxidative stress, leading to effects of histopathological changes in the organs at both intestinal and extraintestinal levels, including in the lung, liver, kidney, and in hematological parameters. Even though aluminum hydroxide is considered safe, our data demonstrated that the long-term use of this compound can cause systemic toxic effects and its indiscriminate use should be avoided.

Considering that aluminum ingestion is the major route by which humans are exposed to aluminum-containing compounds [37], we evaluated the changes caused by long-term ingestion of aluminum hydroxide administration in several organs. After oral administration, about 40% of the ingested aluminum accumulates in the intestinal mucosa, making the intestine the main aluminum store within the body [37]. Our results showed Al ingestion for 6 months causes protein oxidation and lipid peroxidation. Exposure to aluminum appears to increase the production of reactive oxygen species (ROS), which can interact with cellular components and promote damage to lipids and proteins [38]. We have also observed a higher catalase activity in the intestine, which could have occurred to catalyze the decomposition reaction of hydrogen peroxide to water and minimize the oxidative damage in the small intestine. The oxidative damage caused by aluminum hydroxide could lead to intestinal epithelium remodeling. In this regard, previous studies have shown aluminum compounds caused crypt hyperplasia, hyperemia, edema in the lamina propria of the villi, infiltration of inflammatory cells, and necrotic areas in the mucosa [11, 39]. Jeong et al. showed that oral administration of aluminum chloride increased intracellular reactive oxygen species level, thus causing an alteration in the intestinal epithelial integrity [11]. ROS acts as an intracellular signal capable of activating signaling pathways involved with the modulation of inflammatory responses [40]. We observed lower levels of IL-10 in animals exposed to aluminum hydroxide. Low levels of IL-10 have been associated with gastrointestinal tract disease development [41]. Our data suggests that the oral aluminum hydroxide administration induced intestinal epithelium remodeling, causing a shortening of the villi, possibly due to oxidative damage and inflammation. Intestinal remodeling might increase intestinal permeability [11], leading to the systemic effects observed in hematological parameters in the liver, kidneys, and lungs.

In this study, besides the local effects caused by the oral administration of aluminum, our results showed that aluminum hydroxide administration caused lower erythrocyte platelet counts, hematocrit, and hemoglobin compared to control. Although aluminum has low intestinal absorption [42], it promoted changes in hematological parameters when administered for a long period. Also, our results corroborate with Turgut et al. [43] and Ghorbel et al. [9], who have observed lower erythrocyte, hematocrit, and hemoglobin levels in animals given aluminum-containing compounds. It has been described that aluminum intoxication can induce anemia development [13]. However, the molecular mechanisms by which aluminum can exert its toxic effects on hematopoiesis are still unclear [13]. Oxidative stress might be one possible cause involved in aluminum toxicity [37]. Cheng et al. demonstrated in isolated erythrocytes that aluminum caused protein oxidation, leading to cell membrane disruption [38]. Based on our data, we cannot determine which mechanism caused the changes in the hematological parameters; however, it could be possible that oxidative stress could promote anemia in animals exposed to aluminum.

In humans, after oral ingestion, the absorbed aluminum is eliminated by the kidneys and excreted in the urine, though liver excretion, via bile, may also occur [44]. In our study, we observed elevated levels of AST in the group that received aluminum hydroxide. Liver damage, caused by any compound, can be evaluated by measuring aminotransferase levels, where increased levels of these enzymes indicate organ damage [40]. The increases in AST level may be related to the hepatic steatosis that we observed in animals treated with aluminum. Our study demonstrated that hepatic steatosis can impair liver function, promoting an increase in liver enzyme levels [40]. Previous studies have observed disturbances in the lipid profile of aluminum-intoxicated animals caused by an accumulation of the lipids in the liver leading to alterations in lipid metabolism [41, 45]. We detected hepatocyte steatosis in the animals treated with aluminum hydroxide, which corroborates previous findings and demonstrates that exposure to aluminum promotes hepatocyte dysfunction. In addition, we observed increased levels of lipid peroxidation and protein oxidation. Previous studies have shown that the administration of aluminum chloride from 4 to 8 weeks increased lipid peroxidation in rats [45, 46]. The exposition to aluminum, besides promoting oxidative damage, caused a decrease in reduced glutathione and catalase activity [14, 44, 46]. Regarding the altered antioxidant defense, the animals showed lower levels of IL-10. These results correlate with those found in the small intestine and represent an important factor in Al intoxication, as they could make the animals more susceptible to developing other diseases [47].

Long-term aluminum hydroxide ingestion also affects renal function. In our study, we observed reduced urea levels in the plasma. Previous studies observed divergent effects of aluminum intoxication on markers of renal function, as one showed increased urea and uric acid in the plasma [10], and other studies observed no differences for these markers [48, 49]. The liver synthesizes urea from the catabolism of amino acids [50]. Our findings show aluminum exposure impairs liver function possibly reducing protein metabolism, thus decreasing plasma urea levels. Aluminum hydroxide ingestion had a nephrotoxicity effect, as illustrated by the decrease in glomerular density. Previous studies have observed degenerative changes after Al administration, as observed in the present study [48]. An increased ROS production might have caused the changes in renal function observed in our study. Aluminum increased lipid peroxidation and protein oxidation, which could have altered renal physiology and biochemistry [51]. Our results corroborate those found by Ghorbel et al., where the authors observed increased levels of malondialdehyde, carbonylated protein, and advanced oxidation protein product in animals exposed to aluminum chloride for 21 days [10].

Aluminum hydroxide administration induced inflammation and oxidative stress in the lungs, causing lipid peroxidation and protein oxidation, although it did not cause structural changes. Interestingly, in a short-term exposure model, our group showed nebulization with aluminum hydroxide promotes inflammation and oxidative stress in the lungs [18]. The data suggest that regardless of the administration route and time of exposure, aluminum hydroxide exerts toxic effects in the lungs.

Thus, our results show aluminum hydroxide ingestion has systemic toxic effects, suggesting that the long-term use of this compound can have a significant impact on several organs. This observation could be of great importance, as aluminum hydroxide is used in the management and treatment of acid indigestion [16]. Indiscriminate use of this compound could contribute to an increase in the daily consumption of aluminum [52], which can have toxic effects in several organs and systems.

Our study has some limitations. We have not determined the levels of trace elements, such as iron, zinc, and magnesium. These minerals are cofactors of antioxidant enzymes and may have their concentrations altered by aluminum exposure. Also, we did not evaluate the signaling pathways involved in the response triggered by long-term administration of aluminum hydroxide. Further studies are needed to evaluate the mechanisms involved in the response triggered by long-term exposure.

In conclusion, our data indicates that long-term oral administration of aluminum hydroxide promotes toxic effects in the small intestine, liver, kidney, and lung, characterized by inflammation and redox imbalance. Therefore, it might be necessary for the international guidelines, by the international and local regulatory agencies, for aluminum hydroxide consumption to be updated to account for its toxic effects.

## Figures and Tables

**Table 1:** Analysis of hematological parameters in blood and plasma of investigated groups.

	CG	VG	AHG
Erythrocyte (×10^6^/mm^3^)	10.14 ± 0.59	9.76 ± 0.16	9.41 ± 0.17^a^
Hematocrit (%)	46.87 ± 2.00	45.00 ± 0.74	42.65 ± 1.57^a,b^
Hemoglobin (g/dL)	15.47 ± 0.79	15.00 ± 0.25	14.22 ± 0.52^a^
Platelet (×10^6^/mm^3^)	324.60 ± 28.87	293.40 ± 13.83	256.20 ± 18.19^a,b^
Leukocytes (×10^3^/mm^3^)	4.81 ± 2.34	4.98 ± 1.50	4.07 ± 1.51
Monocytes (×10^3^/mm^3^)	0.84 ± 0.46	0.84 ± 0.29	0.48 ± 0.38
Lymphocytes (×10^3^/mm^3^)	1.69 ± 0.90	1.50 ± 0.90	0.98 ± 0.35
Neutrophils (×10^3^/mm^3^)	2.25 ± 1.24	2.60 ± 0.76	2.60 ± 1.05
Eosinophils (×10^3^/mm^3^)	0.02 (0.00–0.06)	0.02 (0.00–0.08)	0.00 (0.00–0.01)
AST (U/L)	110.90 ± 34.30	119.00 ± 20.83	208.80 ± 41.18^a,b^
ALT (U/L)	10.46 ± 3.90	10.62 ± 7.08	7.11 ± 5.55
ALP (U/L)	32.18 ± 6.56	30.14 ± 3.12	35.70 ± 5.72
Gamma-GT (U/L)	4.46 ± 0.80	4.34 ± 1.13	5.24 ± 1.20
Uric acid (mg/dL)	3.90 ± 1.45	2.70 ± 0.19	2.64 ± 0.51
Urea (mg/dL)	46.40 ± 5.14	43.06 ± 1.93	35.76 ± 5.17^a^

CG: control group; VG: vehicle group; AHG: aluminum hydroxide group; ALT: alanine aminotransferase; AST: aspartate aminotransferase; ALP: alkaline phosphatase. The letter (a) represents a significant difference between groups when compared to CG. The letter (b) represents a significant difference between groups when compared to VG. Data were expressed as mean ± standard deviation or median and interval between quartiles and were analyzed by one-way ANOVA or Kruskal-Wallis followed by Tukey or Dunn's posttest, *n* = 6 animals per group.

**Table 2:** Biomarkers of oxidative stress in the lung, liver, kidney and small intestine of investigated groups.

	CG	VG	AHG
Lung			
SOD (U/mg ptn)	20.35 ± 8.55	19.64 ± 5.79	9.91 ± 3.71^a,b^
CAT (U/mg ptn)	3.16 ± 1.59	2.42 ± 1.41	1.21 ± 0.50^a^
GSH/GSSG ratio	10.61 (6.18–12.49)	11.22 (7.27–12.73)	3.76 (2.57–5.47)^a,b^
TBARS (nmol/mg ptn)	0.94 ± 0.10	0.85 ± 0.22	1.47 ± 0.43^a,b^
Carbonyl Protein (nmol/mg ptn)	6.08 ± 4.15	8.46 ± 3.16	20.20 ± 6.73^a,b^
MPO (U/mg ptn)	3.15 ± 1.75	4.73 ± 2.28	6.73 ± 2.71^a^
Liver			
SOD (U/mg ptn)	104.10 ± 30.68	110.90 ± 20.80	98.50 ± 25.45
CAT (U/mg ptn)	0.64 ± 0.29	0.85 ± 0.23	0.30 ± 0.12^a,b^
GSH/GSSG ratio	3.48 (3.01 – 4.24)	2.50 (2.00 – 3.32)	1.60 (1.04 – 3.00) ^a^
TBARS (nmol/mg ptn)	0.56 ± 0.18	0.58 ± 0.10	1.02 ± 0.28^a,b^
Carbonyl Protein (nmol/mg ptn)	6.22 ± 2.24	10.54 ± 3.03	18.52 ± 3.87^a,b^
Kidney			
SOD (U/mg ptn)	5.99 ± 2.13	6.29 ± 0.67	5.48 ± 0.84
CAT (U/mg ptn)	2.33 ± 0.73	1.86 ± 0.27	2.02 ± 0.38
GSH/GSSG ratio	0.46 (0.26–1.11)	1.32 (0.75–1.41)	0.47 (0.12–1.23)
TBARS (nmol/mg ptn)	1.10 ± 0.22	1.11 ± 0.38	1.80 ± 0.60^a,b^
Carbonyl Protein (nmol/mg ptn)	12.02 ± 4.80	14.06 ± 2.36	23.69 ± 5.22^a,b^
Small Intestine			
SOD (U/mg ptn)	7.58 ± 3.18	6.64 ± 5.00	5.45 ± 1.64
CAT (U/mg ptn)	0.33 ± 0.14	0.29 ± 0.84	0.59 ± 0.22^a,b^
TBARS (nmol/mg ptn)	0.57 ± 0.18	0.56 ± 0.16	1.02 ± 0.28^a,b^
Carbonyl Protein (nmol/mg ptn)	11.71 ± 4.42	15.59 ± 4.33	34.19 ± 4.90^a,b^

CG: control group; VG: vehicle group; AHG: aluminum hydroxide group; SOD: superoxide dismutase; CAT: catalase; GSH: glutathione sulfide; GSSG: oxidized glutathione; MPO: myeloperoxidase; TBARS: thiobarbituric acid reactive substances. The letter (a) represents a significant difference between groups when compared to CG. The letter (b) represents a significant difference between groups when compared to VG. Data were expressed as mean ± standard deviation or median and interval between quartiles and were analyzed by one-way ANOVA or Kruskal-Wallis followed by Tukey or Dunn's posttest, *n* = 6 animals per group.

**Table 3:** Inflammatory markers in the lung, liver, kidney, and small intestine of investigated groups.

	CG	VG	AHG
Lung			
CCL2 (pg/mL)	7582.0 ± 4736.98	14244.2 ± 1610.01	17973.0 ± 4843.19^a^
IL-10 (pg/mL)	6470.2 ± 3276.67	6372.4 ± 1312.26	3905.6 ± 1752.64
IL-6 (pg/mL)	4338.6 ± 1208.12	4479.6 ± 1254.95	3124.6 ± 1401.86
Liver			
CCL2 (pg/mL)	26375.4 ± 10402.0	31744.8 ± 8911.17	24995.6 ± 11908.1
IL-10 (pg/mL)	40279.4 ± 10220.3	32664.0 ± 6177.82	23110.4 ± 8896.94^a^
IL-6 (pg/mL)	26546.8 ± 9898.38	26249.4 ± 4951.11	23328.2 ± 9732.51
Kidney			
CCL2 (pg/mL)	14020.4 ± 3898.71	19501.8 ± 4419.22	20769.6 ± 5262.82
IL-10 (pg/mL)	22425.2 ± 2712.15	303044.2 ± 4529.05	23839.8 ± 9236.66
IL-6 (pg/mL)	17940.6 ± 2169.57	24276.0 ± 3623.34	21472.4 ± 7389.61
Small Intestine			
CCL2 (pg/mL)	27208.0 ± 23127.7	15313.0 ± 10334.3	5257.4 ± 2733.3
IL-10 (pg/mL)	22748.4 ± 12900.3	15152.6 ± 10593.8	4346.2 ± 3140.95^a^
IL-6 (pg/mL)	18199.0 ± 10320.6	12122.4 ± 8475.2	3476.8 ± 2512.59^a^

CG: control group; VG: vehicle group; AHG: aluminum hydroxide group; CCL2: monocyte chemotactic protein-1; IL-6: interleukin 6; IL-10: interleukin 10. The letter (a) represents a significant difference between groups when compared to CG. The letter (b) represents a significant difference between groups when compared to VG. Data were expressed as mean ± standard deviation and were analyzed by one-way ANOVA followed by Tukey's posttest, *n* = 6 animals per group.

**Table 4:** Morphometric analysis in the lung, liver, kidney, and small intestine of investigated groups.

	CG	VG	AHG
Lung			
Vv[a] (%)	62.03 (57.19–65.71)	64.69 (61.41–66.80)	62.35 (59.07–70.47)
Vv[sa] (%)	40.47 (37.03–44.14)	35.32 (33.20–38.60)	37.66 (29.53–40.94)
Liver			
Steatosis	0 (0; 0)	0 (0; 0)	4 (4; 4)^a,b^
Inflammatory reaction	0 (0; 0)	0 (0; 0)	0 (0; 0.25)
Kidney			
Glomerular density	0.03 ± 0.006	0.03 ± 0.006	0.01 ± 0.009^a^
Small intestine			
Villus height (*μ*m)	322.97 ± 28.69	330.24 ± 40.32	255.93 ± 44.85^a,b^
Crypt depth (*μ*m)	100.40 ± 25.24	107.26 ± 10.06	123.47 ± 17.60
Vh/Cd ratio	3.20 (3.00; 3.70)	3.30 (2.80; 3.70)	2.10 (1.90; 2.30)^a,b^
Villus area (*μ*m^2^)	34851.2 (33185.4; 37910.4)	31238.0 (26368.3; 33267.9)	24466.3 (22715.2; 26407.7)^a^

CG: control group; VG: vehicle group; AHG: aluminum hydroxide group; Vh: villus height; Cd: crypt depth. The letter (a) represents a significant difference between groups when compared to CG. The letter (b) represents a significant difference between groups when compared to VG. Data were expressed as mean ± standard deviation or median and interval between quartiles and were analyzed by one-way ANOVA or Kruskal-Wallis followed by Tukey or Dunn's posttest, *n* = 6 animals per group.

## Data Availability

Data is available on request.
